# The Central Role of the F-Actin Surface in Myosin Force Generation

**DOI:** 10.3390/biology10121221

**Published:** 2021-11-23

**Authors:** Matthew H. Doran, William Lehman

**Affiliations:** Department of Physiology & Biophysics, Boston University School of Medicine, Boston, MA 02118, USA

**Keywords:** actin, myosin, actin-binding proteins, force production, tropomyosin, molecular motor

## Abstract

**Simple Summary:**

Although actin is a highly conserved protein, it is involved in many diverse cellular processes. Actin owes its diversity of function to its ability to bind to a host of actin-binding proteins (ABPs) that localize across its surface. Among the most studied ABPs is the molecular motor, myosin. Myosin generates force on actin filaments by pairing ATP hydrolysis, product release, and actin-binding to the conformational changes that lead to movement. Central to this process is the progression of myosin binding to the actin surface as it moves through its ATPase cycle. During binding, actin acts as a myosin ATPase activator, catalyzing essential hydrolysis release steps. Here, we use the current model of actin-myosin binding as a roadmap to describe the portions of the actin-myosin interface that are sequentially formed throughout the motor cycle. At each step, we compare the interactions of a diverse set of high-resolution actin-myosin cryo-electron microscopy structures to define what portions of the interface are conserved and which are isoform-specific.

**Abstract:**

Actin is one of the most abundant and versatile proteins in eukaryotic cells. As discussed in many contributions to this Special Issue, its transition from a monomeric G-actin to a filamentous F-actin form plays a critical role in a variety of cellular processes, including control of cell shape and cell motility. Once polymerized from G-actin, F-actin forms the central core of muscle-thin filaments and acts as molecular tracks for myosin-based motor activity. The ATP-dependent cross-bridge cycle of myosin attachment and detachment drives the sliding of myosin thick filaments past thin filaments in muscle and the translocation of cargo in somatic cells. The variation in actin function is dependent on the variation in muscle and non-muscle myosin isoform behavior as well as interactions with a plethora of additional actin-binding proteins. Extensive work has been devoted to defining the kinetics of actin-based force generation powered by the ATPase activity of myosin. In addition, over the past decade, cryo-electron microscopy has revealed the atomic-evel details of the binding of myosin isoforms on the F-actin surface. Most accounts of the structural interactions between myosin and actin are described from the perspective of the myosin molecule. Here, we discuss myosin-binding to actin as viewed from the actin surface. We then describe conserved structural features of actin required for the binding of all or most myosin isoforms while also noting specific interactions unique to myosin isoforms.

## 1. The Role of Actin in the Cell

Actin is derived from a protein superfamily represented among the earliest of cell types [1]. The actin protein sequence is extremely well conserved in eukaryotes, varying by a few amino acids between species and from tissue to tissue [2]. Despite this sequence correspondence, actin participates in a large number of seemingly unrelated cellular processes [3,4,5,6].

Actin’s functional diversity largely depends on the timing of its expression, its localization within a cell, and its interaction with scores of actin-binding proteins (ABPs), which bind either to its monomeric (G-actin) or its filamentous (F-actin) form [7,8,9]. ABPs that target filamentous actin perform a range of functions. For example, cofilin binds to and severs the filament during cytoskeletal remodeling. Alternatively, spectrin acts as an F-actin crosslinking protein, playing a critical role in organizing filaments to maintain cell shape, best illustrated in red blood cells and axons [10,11]. Among the most well-known ABPs is the molecular motor, myosin. Myosin generates force on actin filaments in order to power processes such as vesicle trafficking, membrane deformation, cell movement, and the shortening of sarcomeres in muscle cells [12,13,14,15,16,17,18]. Central to myosin motor activity are structural changes initiated by binding to the F-actin surface.

In this review, we discuss the currently proposed actin-myosin binding mechanism as the myosin motor progresses over the actin surface throughout the motor ATPase cycle. We describe high-resolution cryo-electron microscopy (cryo-EM) structures from a variety of actin-myosin sources to identify the conserved portions of the actin-myosin interface as well as the variable regions that generate isoform-specific, structurally-distinct binding.

### The Topology of the Actin Filament

**Figure 1:**
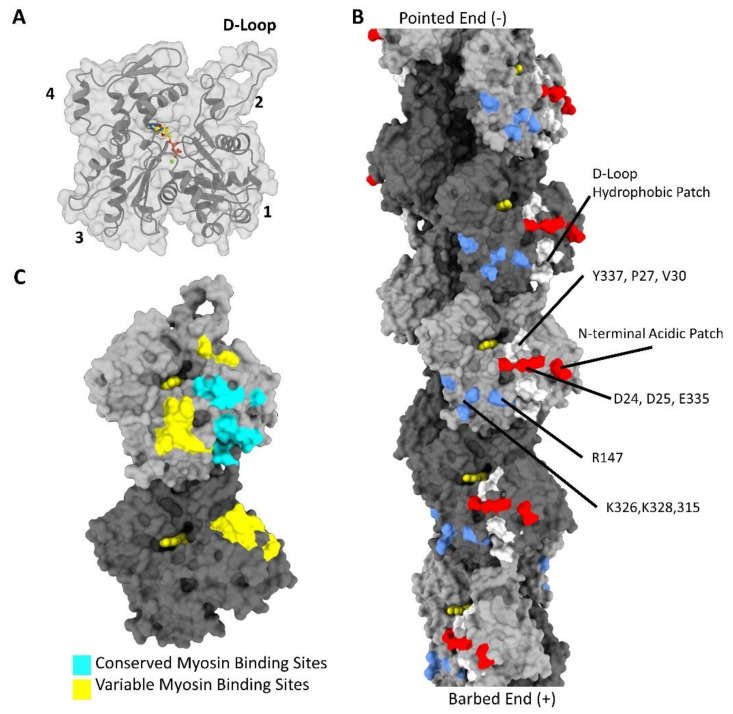
Topology of the actin filament. The surface of actin is targeted by numerous proteins using unique binding sites. (**A**). Organization of an F-actin subunit. The subunit secondary structure and surface are visible with the subdomains numbered and the DNase binding loop (D-loop) indicated. ADP-Mg^2+^ is shown in color in the center of the molecule within the nucleotide-binding cleft. (**B**). Survey of the actin surface. Particular regions of the actin surface used by various F-actin-binding proteins are shown. Hydrophobic surfaces in white, acidic surfaces in red, and basic surfaces in blue. The ADP is shown in the center of each subunit in yellow. Alternating subunits are shaded dark and light grey. (**C**). Conserved and variable actin-myosin binding sites. This figure displays the binding surface of myosin at the junction of two actin subunits. In cyan is the conserved myosin-binding site, which is found in all six myosin isoforms compared in this review. These sites are comprised of the subdomain 1-HLH motif interface, subdomain 1-CM-loop interface, and the N-Terminus-Loop 2 interface. The yellow regions demarcate the regions of the binding site where the interactions between isoforms are not conserved. This variable region is made up of the actin regions that contact myosin loop 3, loop 4, the activation loop, and the tip of the CM loop.

The F-actin subunit has four subdomains with a nucleotide-binding site located at the center of the molecule, as shown in Figure 1A [2]. During the transition from monomeric G-actin to filamentous F-actin, the exposed surface of the molecule changes considerably [19,20,21]. Among these changes is the flattening of the actin subunit, which involves a 20-degree rotation around an axis running through subdomains 1 and 3. Subunit flattening promotes the interactions needed for helical polymerization [20]. The resulting filament has two strands winding around one another to form a long-pitch right-handed helix, while at the same time subunits on opposite strands interact to form a short-pitch left-handed helix [21]. Actin subunits are also arranged in a polar manner with the edges of subdomains 1 and 3 facing toward the barbed/plus end of the filament and subdomains 2 and 4 facing the opposite pointed/minus end. After polymerization, the resulting face of the actin filament remains exposed to solvent and available for ABPs [20,22].

The ABP-accessible sites on F-actin involve both electrostatic and hydrophobic regions distributed widely over the filament surface (Figure 1B). For example, a patch of positively charged residues on subdomain 3 containing residues K315, K326, K328, and R147 plays a major role in binding to tropomyosin [23]. An acidic patch is also found on subdomain 1, formed by the actin N-terminus as well as the adjacent residues E334, D24, and D25, which are involved in the binding of several ABPs. Besides surface electrostatic residues, several clusters of non-polar amino acids are also exposed to solvent. One such hydrophobic surface contains residues Y337, P27, and V30, which interact with myosin [24]. A second hydrophobic patch sometimes referred to as a groove, appears at the junction between intrastrand subunits comprised of residues M44 and M47 from the DNase-binding loop (D-loop) (see Figure 1B) and residues Y143, L349, I345, and I341 from the subdomain 1 and 3 of the minus end side of an adjacent subunit [25]. This hydrophobic patch plays an important role in actin polymerization, acts as a target for post-translational modifications (PTMs), and is a locus for the binding of ABPs (see Supplementary Item Table S1) [26,27,28,29].

Many ABPs bind to the D-loop hydrophobic patch by exploiting their calponin homology (CH) domain, a conserved actin-binding domain [30,31,32]. The CH domain-containing proteins α-actinin, spectrin, dystrophin, utrophin, filamin, and plastin, bind at this site regardless of their diverse cellular function [33,34,35,36,37,38]. Other ABPs lacking CH domains also target this region. Lifeact, for example, a small peptide that has become a gold standard for cell imaging of actin structures, binds to the same hydrophobic patch, competing with proteins that contain the CH domain [29,33]. Binding interactions of cofilin and myosin, two more CH domain-free proteins, also involve this region [29]. Although many F-actin-binding proteins use this hydrophobic patch, individual ABPs have their unique binding footprints that exploit additional distinctive surface residues. Furthermore, though ABPs may target similar regions of the filament, they can differentially modulate actin filament dynamics through differences in binding modes, using functional domains that are distinct from their actin-binding domains, or developing binding site adaptations that suit their function, as seen in leiomodin and tropomodulin [39].

One ABP example that uses the D-loop hydrophobic patch is the CH domain-free vinculin, which plays an essential structural role in cytoskeletal focal adhesions and adherens junctions helping to link actin to the extracellular matrix [40,41]. Vinculin’s actin-binding domain contains a four-helix bundle and an elongated C-terminal extension [42]. Although the four-helix bundle makes hydrophobic contacts at the D-loop hydrophobic site, the bundle also extends towards subdomain 3, where it twists along the contour of the actin surface to make a series of electrostatic contacts. Additionally, the C-terminal extension extends over subdomain 1, forming multiple unique hydrophobic and electrostatic interactions [43].

In contrast, several other ABPs, such as N-terminal domains of myosin binding protein C, myosin light chain kinase, and tropomyosin, do not bind to the aforementioned hydrophobic junction between actin subunits [44,45,46,47]. For example, tropomyosin uses its long coiled-coil to bind continuously over many successive actin subunits through repeating actin-binding sequences [48]. Each tropomyosin pseudo-repeat binds over the border between actin subdomains 3 and 4, largely through periodic electrostatic interactions on the surface of the filament [22,49]. There are over 40 mammalian tropomyosin isoforms generated from 4 alternatively spliced genes that are thought to play a pivotal role in limiting or specifying the binding of ABPs, thereby further defining functions of intracellular actin filaments [50,51]. Moreover, in striated muscle sarcomeres, tropomyosin, in conjunction with troponin, sterically regulates myosin binding to the actin surface [52,53,54]. As shown in Figure 1B, the F-actin surface displays a diverse set of residues that enable the selective binding of a variety of proteins.

In addition to the binding of many ABPs, over 100 post-translational modifications of the actin filament have been identified, which regulate ABP binding as well as filament topology and dynamics [55]. One classic example of an actin post-translational modification affecting filament dynamics is the oxidation of the D-loop residues M44 and M47 by the MICAL enzymes. These enzymes bind to actin and catalyze the oxidation of methionine, which causes depolymerization as well as the recruitment of the F-actin remodeling ABP, cofilin [56]. The conversion of the methionine residues at positions 44 and 47 to their oxidized form most likely destabilizes the F-actin D-loop conformation, leading to the destabilization of the filament [57]. The binding of ABPs as well as the regulation of filament dynamics by post-translational modifications enables actin to be involved in diverse cell functions.

The actin filament is perhaps best known for its role in force generation. Here, the F-actin surface, as outlined above, acts as a molecular track for myosin motors [12,13,14]. In myosin-based motility, force generation relies on actin-myosin binding coupled with myosin ATP hydrolysis [58]. Here, the actin surface catalyzes myosin ATPase activity by sequentially binding to myosin subdomains, inducing conformational changes within the motor, and initiating hydrolysis product release. The topology of the actin filament and its dynamic interaction with myosin is central to the timing of these critical steps that lead to actomyosin force and motion generation.

## 2. Introduction to Myosin Structure

**Figure 2:**
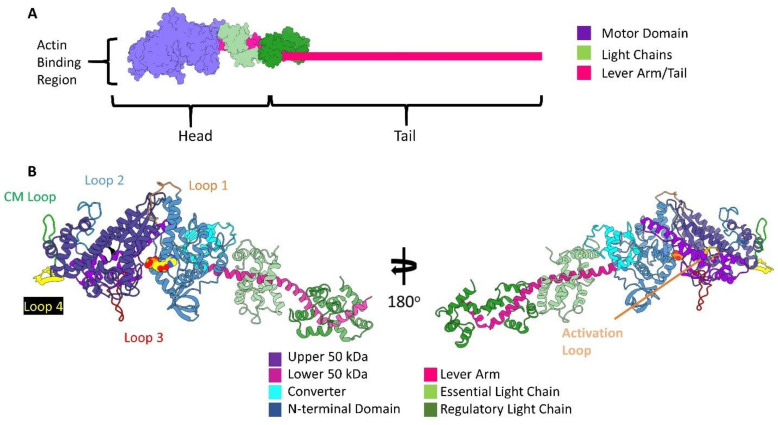
Structural organization of the myosin molecule. (**A**). Classical organization. The motor domain is colored in purple, the essential light chain in light green, the regulatory light chain in dark green, and the lever arm, which extends to the tail in pink. The actin-binding region is found in the motor domain. (**B**). Ribbon diagram of the myosin motor domain organization in two orientations. The secondary structure representation of the rigor skeletal muscle myosin II (PDB:5H53) [74] is colored by myosin subdomains. The surface loops of the molecule are also shown. Lastly, the nucleotide-binding site is specified with an ADP space-filling molecule. All figures rendered in ChimeraX.

Although myosin motors translocate along F-actin with different speeds and forces, the basic unit of the molecule is conserved and well described [59,60]. Myosin contains two structurally distinct regions called the head and tail (Figure 2A). In all myosin classes, the head domain contains the actin-binding site and ATPase activity that is sufficient to produce force and movement. Within the head, or S1 fragment is the motor domain and the lever arm, which undergo conformational changes during force production in conjunction with ATP hydrolysis [61,62,63]. The entire head apparatus is linked to an elongated tail domain. The tail domain is responsible for myosin coiled-coil dimerization and the bivalent nature of most myosins. These domains are also isoform-specific and can associate with various distinct cellular cargo, membrane-spanning motifs, as well as forming the shaft of the thick filament [64,65,66,67].

The motor domain, of greatest interest here, is further split into four distinct subdomains: the actin-binding upper and lower 50 kDa domains, the N-terminal domain, and the mobile converter domain with the associated lever arm see Figure 2B. In addition to the four subdomains are surface loops that extend from the core of the motor domain, called the cardiomyopathy loop (CM loop), loops 1, 2, 3, and 4. The CM loop was initially identified as a hotspot for disease-related missense mutations (at position R403) leading to familial hypertrophic cardiomyopathy [68,69,70,71,72,73].

The myosin nucleotide-binding site is far from the actin-binding region, positioned between a seven-stranded β-sheet in the upper 50 kDa domain called the transducer and the N-terminal domain [75]. Still, progressive changes during actin-myosin binding induce hydrolysis and product release to trigger large-scale myosin conformational changes that lead to lever arm rotation and force production [76].

**Figure 3:**
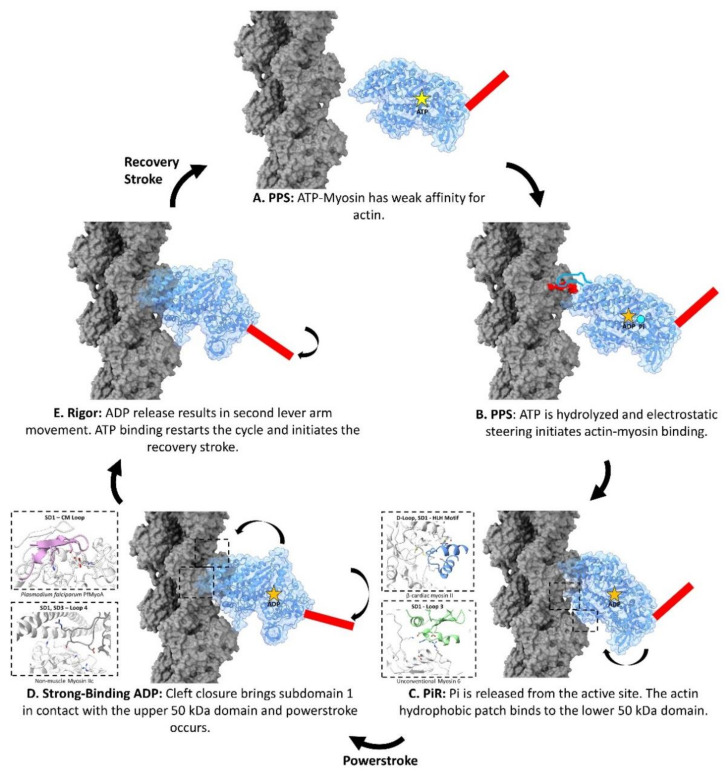
Proposed binding mechanism of the actin-myosin complex. The myosin motor domain structural rearrangements are depicted for each step of the motor cycle. The surface of myosin in transparent blue shows the secondary structure of the protein. The lever arm is indicated as a red bar. The surface of the actin filament is depicted in grey. (**A**). Pre-powerstroke state (PPS) myosin with bound ATP (yellow star) has a very weak affinity to the actin surface. The cleft between the upper and lower 50 kDa domains is open and the lever arm is in the primed pre-powerstroke state conformation. This PPS state is depicted using the PDB: 5N6A [82]. (**B**). PPS myosin hydrolyzes ATP to ADP-Pi. Electrostatic steering between the acidic patches on the actin surface and myosin surface loop 2 initiates binding between actin and myosin. The acidic patch of actin subdomain 1, containing the N-terminus of actin as well as residues D24, D25, and E334 are indicated in red on the actin filament. Loop 2 of myosin is depicted as a cartoon (blue), while the ADP and Pi molecules are depicted as an orange star and cyan circle respectively. This PPS state is depicted using the PDB: 5N6A [82]. (**C**). Pi is released from the active site to create the PiR intermediate state. According to the currently proposed binding mechanism, the hydrophobic patch at the junction of actin subunits binds to the lower 50 kDa domain HLH motif. Loop 3 and the activation loop are also brought to the actin surface. The insets describe the binding of the β-cardiac myosin II isoform (PDB: 6X5Z, 7JH7) [83,84] HLH motif and unconventional myosin 6 (PDB: 6BNP) [85] loop 3 as examples. The PiR state is depicted using the PDB: 4PFO [72]. (**D**). The powerstroke and cleft closure occurs producing the strong-binding ADP state. The lever arm undergoes its major swing, the powerstroke. Total cleft closure is also induced by stabilization of the strut and the binding of the upper 50 kDa domain to actin subdomain 1. Upper 50 kDa binding is accomplished through conserved hydrophobic contacts between actin subdomain 1 and the CM loop. Myosin loop 4 is also brought close to actin-tropomyosin. The insets describe the binding of the PfMyoA CM loop and the NM2c loop 4 are shown as examples. The strong binding actin-myosin-ADP motor domain is depicted using the PDB: 6BNQ [85]. (**E**). ADP release yields the rigor conformation. Along with the release of ADP from the active site, the lever arm goes through another, yet a smaller movement. This is the last stage of the cycle, where ATP re-binding triggers cleft re-opening and release of myosin from the actin surface. The lever arm rotates back to its pre-powerstroke state conformation in a process called the recovery stroke. The rigor-actin complex is depicted using the PDB: 6X5Z [83].

Despite varying functional requirements, biochemical and structural data show that all myosin motors use the same fundamental sequence of steps to generate a force on actin, (see Figure 3) [58,77,78]. In the first step, the pre-powerstroke state (PPS) myosin-ATP-Mg^2+^ complex has a weak affinity for the F-actin surface. Once myosin catalyzes ATP, the PPS myosin-ADP-Pi-Mg^2+^ is thought to bind weakly to the actin surface through electrostatic interactions.

After contact with actin is initiated, inorganic phosphate is released from the nucleotide-binding site, forming the Pi release state (PiR state) [77]. As the PiR state myosin progresses through its binding interaction on the actin surface, the upper and lower 50 kDa domains rotate toward one another, closing the intervening cleft. Cleft closure ensures that the actin-binding regions of the myosin head orient themselves to their respective spots on the actin surface [76,79]. Upon cleft closure, the lever arm swings in a process dubbed the “powerstroke”, generating a large amount of force on the filament and leaving the myosin-ADP-Mg^2+^ complex anchored to the actin surface. Next, ADP-Mg^2+^ is released from myosin and the lever arm goes through another, albeit smaller, movement to form the nucleotide-free rigor conformation [80]. The force generation cycle restarts once ATP rebinds to the rigor myosin and is released from actin. After release from actin, the upper and lower 50 kDa domains move away from each other, and the lever arm is rotated back to its original PPS position in a movement called the “recovery stroke” [81].

## 3. The Sequential Binding of Myosin to Actin

Vital for this force-generating process is the binding of myosin to the actin surface during the motor’s ATPase cycle. To understand the interactions responsible for motor activity, the detailed changes between the actin surface and the myosin head as the motor progresses through this cycle must be defined. Results inferred from X-ray crystallography and high-resolution cryo-EM experiments make this possible [24,77].

In the following sections, we use current views of the proposed actin-myosin binding mechanism as a guide to describing the portions of the interface that are consecutively formed during the kinetic cycle. At each of these sites, we compare the residue-level interactions from a set of six actin-myosin complexes that vary both functionally and according to tissue and species origin. Our comparisons rely on the contacts of the actin-myosin complexes in the rigor or ADP-bound form available in recent high-resolution cryo-EM structures described in Supplementary Item Table S2 [24,74,80,83,84,85,86,87,88]. 

Our analysis agrees with previous actin-myosin comparisons that indicate myosin binds to two distinct actin interface regions, as reported in ref. [88]. First, a conserved interface is noted and found in all F-actin-myosin cryo-EM structures published to date. These interactions play a vital role in the actin-myosin binding mechanism. The second, more variable interface, creates a unique isoform-specific footprint on the actin surface. Importantly, disease-causing mutations are frequently found at these interfaces, as they most likely disrupt proper actin-myosin binding. In the following sections, we discuss these actin-myosin binding sites in detail as they are formed during the actin-myosin ATPase cycle. We establish which sites are conserved and identify features of the interface that are unique to specific isoforms (highlighted diagrammatically in Figure 1C). We use the amino acid numbering of the cardiac actin-myosin contacts unless otherwise stated.

## 4. Electrostatic Steering Initiates Actin-Myosin Binding

### 4.1. Conserved Actin-Loop 2 Interactions

At the beginning of the ATPase cycle, the pre-powerstroke state myosin has a very weak affinity for the actin filament (see Figure 3A) [82]. As shown in Figure 3B, it is hypothesized that the charged actin N-terminus and close-by acidic residues on subdomains 1 make transient contacts with the basic loop 2 of the PPS myosin during these initial stages of the ATPase cycle [24,89,90,91]. Here, electrostatic steering, a process involving complementary charged surfaces, establishes the initial weak binding between myosin and the actin surface.

### 4.2. Isoform-Specific Actin-Loop 2 Interactions

Although the interaction between the acidic residues on subdomain 1 and the myosin loop 2 is most likely involved in the initial binding event for all myosin motors, loop 2 is also the most variable part of the myosin head and its length can differ by over 100 residues [92]. These differences have dramatic effects on actin-binding. Small loop 2 inserts, as seen in *Plasmodium falciparum* myosin motor, PfMyoA, as well as the mammalian unconventional myosin 6, and myosin 1b, exclude actin electrostatic interactions beyond conserved lysine residues. Longer myosin loop 2 s, however, are thought to form significant interfaces with the actin surface. This is best demonstrated by the myosin IX isoform, which is a single-headed signaling motor that continuously moves along the actin filament [93]. This isoform has an insert spanning 110 to 145 amino acids that are thought to play a role as an electrostatic tether that keeps the motor localized to the actin surface even in the weak-affinity ATP-bound PPS [94,95]. By keeping the motor close to the actin, this elongated loop 2 enables myosin IX to act as a processive myosin, continuously traveling along the actin track and rebinding to its surface as it progresses through its ATPase cycle.

Further biochemical evidence from studies on myosin V has shown that in addition to the size of the loop, the net charge also plays a role in the motor’s affinity for the actin surface [96]. Here, larger net positive charges increase actin affinity in all nucleotide states. Thus, the highly charged but relatively short myosin V loop 2 allows it to move processively, advancing along actin by keeping the motor domain close to its actin acidic patches following detachment [96]. These examples illustrate that the variable nature of the actin-loop 2 interface can vastly change myosin-binding properties and impact its physiological role. Specifically, the interaction between the acidic actin patch on subdomain 1, containing the N-terminus, and myosin loop 2 confines the motor to the actin surface to promote the binding of the next set of actin interfaces.

## 5. Actin D-Loop Hydrophobic Patch Binds to the PiR State Lower 50 kDa Domain

### 5.1. The D-Loop Hydrophobic Patch Makes Conserved Contacts with the Hydrophobic HLH Motif

After the electrostatic steering between the actin N-terminus/subdomain 1 and the myosin surface loop 2 initiates binding and Pi release, the D-loop hydrophobic patch between adjacent actin subunits prompts the binding of the myosin helix-loop-helix (HLH) motif as visualized in Figure 3C. This hydrophobic interaction is a central feature in all known myosin isoforms [77,97,98,99]. Notably, this hydrophobic site on actin is used by other ABPs, particularly ones that contain CH-domains [29]. This interaction site represents the largest interface between the actin filament and myosin, as demonstrated by buried surface calculations in Supplementary Table S3, hence making it a key component of the conserved interface. 

**Figure 4:**
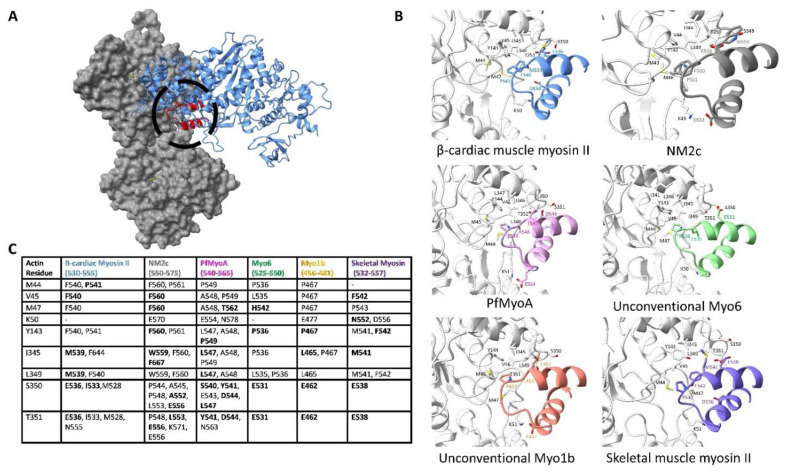
The actin D-loop hydrophobic patch buries the myosin HLH motif. (**A**). The basic positioning of the actin surface relative to the HLH motif in the motor domain. The motor domain is in blue while the HLH motif is colored in red and circled; actin is gray. (**B**). The residue-level binding interfaces between the actin hydrophobic patch surrounding the D loop and myosin HLH motif from the six different structures that are indicated in Supplementary Item Table S2. Because 6X5Z and 7JH7 are both cardiac β-myosin II isoforms, only one is shown for comparisons in the following figures. Actin backbone and residues are colored white, while myosin residues are colored by isoform. Noted residues involved in binding are shown and labeled. These types of depictions were made by extracting the central 2 actin subunits and 1 myosin from each cryo-EM structure and marking relevant residues involved in binding. It is apparent in these figures that the binding mode of the HLH motif is conserved in each myosin isoform. Conserved hydrophobic residues of the actin surface make contact with the HLH motif, while additional electrostatic interactions contribute to binding. (**C**). Residue-to-residue contacts determined for the cryo-EM structures indicated in Supplementary Item Table S2. All residues on myosin that come within 5 Å of the indicated actin residue are listed with residues coming within 4 Å appearing in bold. The actin sequence is based on the Chicken ACTA1 numbering (UniProt: P68139), which is within 1 residue of the other actin isoforms used in the six cryo-EM studies.

The myosin HLH motif is organized as an N-terminal helix, followed by a five-residue loop, and a C-terminal helix. Conserved actin residues L349, I345, and Y143 of subdomains 1 and 3 as well as residues M47, V45, and M44 from the D-loop enclose the HLH motif in all myosin isoforms compared in Figure 4. This hydrophobic patch is thought to resemble a “lock” penetrated by the HLH motif “key”. Here, several hydrophobic residues of myosin, particularly F540 and P541, form close interactions with the actin surface. These interactions are crucial to actin-myosin binding as shown in in vitro studies, where mutating either residue to alanine completely abolishes or severely decreases actin-based motility [91]. The importance of this region is also reflected by the occurrence of disease-causing mutations that are found at this interface. For example, in the cardiac β-myosin II isoform, mutations at I533 and M539 are known to cause cardiomyopathies [84,100].

Apart from the hydrophobic interactions between F-actin and the HLH motif, several conserved polar residues are involved in the actin-myosin interactions. One example is between the backbone of residues T351 and S350 on actin subdomain 1, which make conserved interactions with myosin acidic residues at position 536 on the N-terminal helix. This close interaction, which is frequently within 3 Å, is conserved in nearly all myosin isoforms [24]. Kinetic studies have shown that mutating this residue in *Dictyostelium discoideum* myosin II (E531Q) reduces actin affinity by a factor of ten, demonstrating its importance [89].

Additional electrostatic interactions are found between the C-terminal helix and a conserved D-loop lysine residue. For instance, in the human cytoplasmic non-muscle myosin IIc (NM2c) structure, K49 of the D-loop interacts with myosin residue E550. Similar interactions are formed in all other myosin isoforms shown in Figure 4 except skeletal muscle myosin II. In addition to hydrophobic interactions, these polar interactions further secure the HLH motif and the lower 50 kDa domain to the actin surface.

Much like other ABPs, the hydrophobic myosin HLH motif fits directly into the hydrophobic patch near the D-loop on F-actin, forming a crucial part of the conserved interface during PiR binding. K_d_ measurements, shown in Supplementary Table S3, indicate that the actin-HLH motif is the strongest actin-myosin interface, likely anchoring the complex during PiR binding. Succeeding the binding of the lower 50 kDa domain to the actin surface, the resulting actin-myosin complex is primed to promote cleft closure by the rotation of the upper 50 kDa domain to the actin surface in the next progression of the motor cycle.

### 5.2. Subdomain 1 Makes Isoform-Specific Contacts with Loop 3 upon Lower 50 kDa Binding

**Figure 5:**
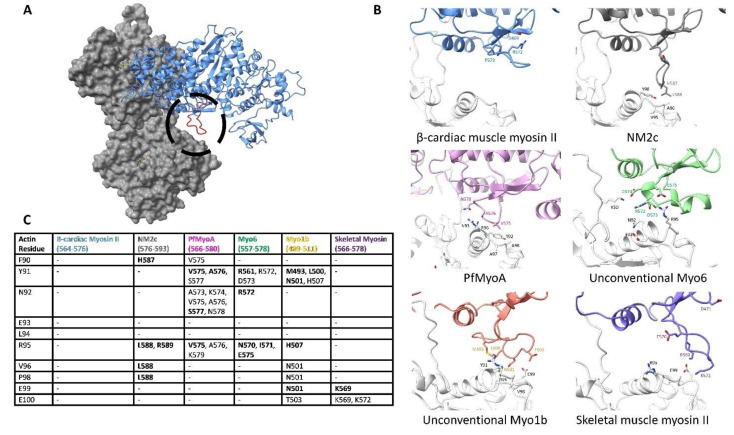
Actin subdomain 1 contacts myosin loop 3. (**A**). The overall positioning of the actin surface relative to loop 3. The motor domain is in blue while loop 3 is colored in red and circled; actin is in gray. (**B**). The residue-level binding interface between actin subdomain 1 and myosin loop 3 from the myosin structures is indicated in Supplementary Item Table S2. Actin backbone and residues are colored white, while myosin residues are colored by isoform. Noted residues involved in binding are shown and labeled. This figure makes it clear that loop 3 is variable and makes isoform-specific contacts with the actin subdomain 1. The cardiac β-myosin II makes no contact with the actin surface, while unconventional myosin 6 and 1b make a series of interactions. (**C**). Residue-to-residue contacts determined for the cryo-EM structures indicated in Supplementary Item Table S2. All residues on myosin that come within 5 Å of the indicated actin residue are listed with residues coming within 4 Å appearing in bold. The actin sequence is based on the Chicken ACTA1 numbering (UniProt: P68139). The myosin II isoforms make significantly fewer interactions compared with other myosins.

Although the binding of the F-actin hydrophobic patch to the myosin HLH motif forms a key portion of the conserved actin-myosin interface during the PiR state, the binding of the lower 50 kDa domain would also bring the variable surface loop 3 close to the actin surface. In fact, in most of the cryo-EM structures, actin subdomain 1 on the adjacent actin subunit, as shown in Figure 3C, comes into close contact with myosin loop 3 in an interaction originally named the Milligan’s contact. This contact was predicted to form based on early structure work and the complementary charged nature of the basic loop 3 with the acidic residues on actin subdomain 1 [101,102]. Due to its highly variable nature, different myosin isoforms from vastly different loop 3-actin interfaces, which are described in detail in Figure 5.

Isoforms with a short loop 3 make only a small footprint on the subdomain 1 surface, while isoforms with much longer loops can form an extensive interface [98,101,103]. For example, loop 3 in the NM2c isoform is relatively short and is oriented in a down position such that the actin helix 79–94 and the adjacent loop make contact with only a few amino acids at the tip of the loop. Conversely, the similarly sized loop 3 in cardiac β-myosin II faces the body of the myosin lower 50 kDa, disallowing any significant interaction with the actin surface.

Other myosins with a long loop 3 can make extensive contacts with the actin surface. For instance, the unconventional myosin 6 loop 3 contains 20 amino acids with two small helices, forming a sizeable footprint on actin subdomain 1. Actin residues N92, E93, and N95 come into close contact with myosin N570, R572, E575, creating a large complementary face. The variability in the size of loop 3 greatly changes the actin surface it can contact, potentially altering the actin surface explored during the PiR state. Isoforms with a long loop 3 may have an increased affinity in the PiR state or these variable interactions may alter the binding pose on the actin surface. It remains to be seen how the variable interactions between actin subdomain 1 and loop 3 impact the binding and kinetics of actin-myosin isoforms.

### 5.3. Subdomain 1 Binds to the Activation Loop

Actin subdomain 1 can also make contact with a variable myosin loop termed the activation loop. The binding of actin subdomain 1 to the activation loop may play a role in activating myosin heads or orienting the lower 50 kDa domain on actin [104,105]. The importance of the loop is controversial and it has alternatively been called the “supporting loop” [24]. The main isoform differences are in the sizes of the loop. The smallest loop, as seen in the β-cardiac and skeletal myosin II isoforms, only contains 4 residues that face actin subdomain 1. Longer loops, such as the 8-residue loop in the NM2c structure, may interact with the N-terminus of actin, but no direct electrostatic interactions are formed. Although biochemical and in vivo analysis has shown that this region increases the number of active myosin heads, it appears to play only a minor role in actin-myosin binding [105].

## 6. The Actin Surface Prompts Myosin Cleft Closure

### 6.1. The N-Terminus of Actin and the C-Terminal Base of Loop 2 Initiate Cleft Closure

The interaction between the acidic patches of actin subdomain 1 and myosin loop 2 is not only important in the initial binding event between the two proteins but is also proposed to be involved in the mechanism of myosin cleft closure [24]. Here, the acidic N-terminus of actin interacts with the basic C-terminus of myosin loop 2. The stabilized C-terminus of loop 2 then binds to the strut, an acidic loop that connects the upper and lowers 50 kDa domains. By binding to loop 2, the strut appears to stabilize the cleft-closed 50 kDa domain conformation [106,107].

In NM2c, for example, the N-terminal residues of actin D1-3 stabilize the loop 2 residues R661 and R663, which moves the strut from its open-cleft PPS conformation to its closed-cleft position [24]. These C-terminal loop 2 residues are also visible in β-cardiac myosin II isoform, where the actin loop, containing residues D24 and D25, is directly opposite myosin residue K639, while acidic actin residues at the N-terminus would face the myosin residue K640 [84]. Additionally, the removal of these two basic residues in myosin II isoforms has been shown to block the ability of myosin to undergo the weak to strong transition [90]. These structural and biochemical data demonstrate that the acidic patch on actin subdomain 1 participates in dual roles in myosin binding. The first initiates myosin-binding while the second helps stabilize the closed cleft conformation of the upper and lower 50 kDa domains.

### 6.2. Cleft Closure Results in F-Actin Subdomain 1 Interacting with the CM Loop 

**Figure 6:**
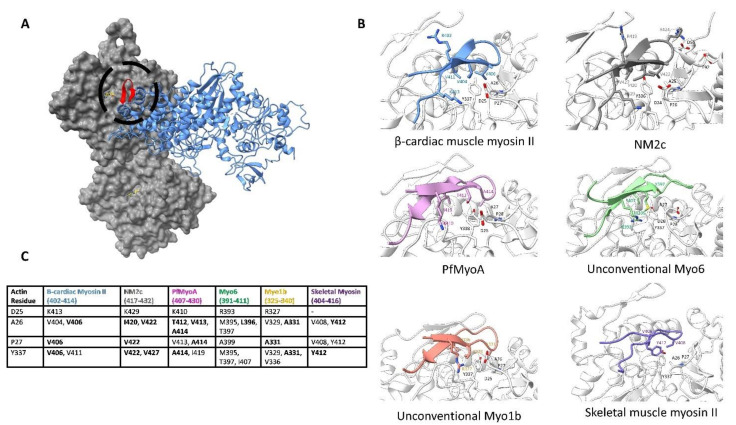
F-actin subdomain 1 binds to the CM loop. (**A**). The overall positioning of the actin surface relative to the CM loop. The motor domain is in blue while the CM loop is colored in red and circled; actin is in gray. (**B**). The residue-level binding interface between actin subdomain 1 and the myosin CM loop from the six myosin structures is indicated in Supplementary Item Table S2. Actin backbone and residues are colored white, while myosin residues are colored by isoform. Noted residues involved in binding are shown and labeled. In each isoform, the hydrophobic actin residues face at least three apolar myosin residues. Additional electrostatic interactions at the base of the loop play a further role in binding. (**C**). Residue-to-residue contacts determined for the cryo-EM structures indicated in Supplementary Item Table S2. All residues on myosin that come within 5 Å of the indicated actin residue are listed with residues coming within 4 Å appearing in bold. The actin sequence is based on the Chicken ACTA1 numbering (UniProt: P68139). The extent of the hydrophobic surface between subdomain 1 and the CM loop is clear when looking at the contacts of individual isoforms.

In addition to the stabilization of the strut, cleft closure is further promoted by hydrophobic residues on actin subdomain 1, which bind to the upper 50 kDa domain as shown in Figure 3D. Here, conserved actin hydrophobic residues bind to the CM loop. For example, in the cardiac β-myosin II isoform, actin residues Y337, I341, P27, and A26 face myosin, creating a hydrophobic surface for the CM loop residues to interact with. In this myosin isoform, three valine residues 404, 406, and 411 on the CM loop bury the actin hydrophobic surface. These are likely to be mechanistically important since mutations at each of these three residues have been found to cause hypertrophic cardiomyopathy [108,109]. Similarly, in the NM2c structure, residues V427, I420, and V422 form a hydrophobic face opposite to the actin surface. The same conserved actin hydrophobic residues interact with at least three CM loop hydrophobic residues in all isoforms of myosin we compare in Figure 6.

Apart from these hydrophobic interactions, the actin surface also provides stabilizing electrostatic interactions. For instance, a salt bridge is formed between the actin residue D25 with myosin residue R410 in the unconventional myosin 1b isoform. Similar electrostatic interactions are seen in the other isoforms. It is clear in Figure 6 that although the specific interactions are divergent in different myosin motors, the position of the CM loop on the actin surface is conserved. 

At the tip of the loop, however, myosin sequences differ by length and charge. This is best illustrated through NM2c and myosin 1b, where actin residues D55 and E92 interact with charged myosin residues. Therefore, although the tip of the CM loop constitutes a small portion of the variable interface, it is striking how actin constrains all CM-loops in a conserved manner. 

### 6.3. Actin Subdomain 3 Binds to Loop 4 to Form the Edge of the Actin-Myosin Interface

**Figure 7:**
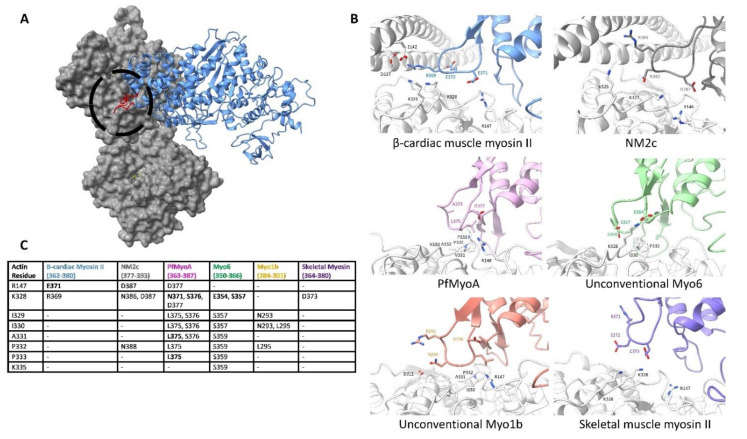
Actin subdomain 3 binds to myosin loop 4 to form the edge of the actin-myosin interface. (**A**). The overall positioning of the actin surface relative to myosin loop 4. The motor domain is in blue while loop 4 is colored in red and circled; actin is in gray. (**B**). The residue-level binding interface between actin subdomain 3, tropomyosin, and myosin loop 4 from the myosin structures is indicated in Supplementary Item Table S2. Actin backbone and residues are colored white, while myosin residues are colored by isoform. Noted residues involved in binding are shown and labeled. The presence of tropomyosin impacts the interactions between actin and myosin. In isoforms that interact with tropomyosin, like cardiac β-myosin II, the residues at the tip of the loop interact with the coiled-coil of tropomyosin. Without tropomyosin, the tip of the loop can reach across to actin subdomain 3, as seen in the unconventional myosin 1b. (**C**). Residue-to-residue contacts determined for the cryo-EM structures indicated in Supplementary Item Table S2. All residues on myosin that come within 5 Å of the indicated actin residue are listed with residues coming within 4 Å appearing in bold. The actin sequence is based on the Chicken ACTA1 numbering (UniProt: P68139).

Cleft closure not only brings the CM loop into contact with the actin surface but also brings the upper 50 kDa domain myosin loop 4 into contact with actin-tropomyosin, which is shown in Figure 3D. The interface between actin and myosin loop 4 forms the outer boundary of the myosin-binding footprint, extending past subdomain 1 to contact the edge of subdomain 3 as shown in detail in Figure 7. This is illustrated in the myosin 1b structure, where the actin subdomain 3 residues I329, I330, and P332 form hydrophobic contacts with the myosin 1b loop 4 residue L295. In addition to hydrophobic contacts, actin electrostatic residues are involved in the striated skeletal myosin II, cardiac muscle myosin II, and in the NM2c isoforms, where actin residues R147 and K328 face cardiac myosin residue E371. Mutations at this site (MYH3 residue 375) in the embryonic skeletal muscle isoform, MYH3, cause Freeman-Sheldon syndrome [72]. Although the actin-loop 4 interface is fairly small compared to other actin-contacting regions, loop 4 plays an important regulatory role due to its interaction with tropomyosin [83,110,111].

Almost all actin filaments are decorated with tropomyosin, which is differentiated by sequence, localization, and timing of expression [51]. In our recent publication, we paired our 4.2 Å cryo-EM structure of the actin-tropomyosin-cardiac myosin II with protein-protein docking techniques to show that favorable interactions between myosin loop 4 and residues on tropomyosin stabilize the contraction-activated tropomyosin position [83]. Similar regulatory schemes may extend to other F-actin filaments, where tropomyosin isoforms modulate the actin-binding activity of specific ABPs. Unconventional myosin 1b, for example, fails to bind to tropomyosin-bound F-actin. Of note, actin-based binding and in vitro motility are rescued after replacing the myosin 1b loop 4 with the *Dictyostelium discoideum* loop 4 [111]. This suggests that loop 4 may be an essential component of tropomyosin-based actomyosin regulation. Thus, by altering the actin surface, tropomyosin isoforms create specialized filaments that have unique roles in the cell [1,49,112,113]. Studying the effects of tropomyosin-bound actin filaments and their interactions with myosin motors and other ABPs is crucial in understanding actin’s diverse functions.

## 7. Conclusions and Perspectives

Throughout the ATPase cycle, the actin surface defines how myosin progresses from the weak binding PPS to the rigor complex. Here, actin acts as a myosin ATPase activator, pairing sequential actin-myosin binding with ATP hydrolysis and product release. Subsequent force is produced through the conformational changes in the myosin motor domain, notably in the lever arm. In order to define the actin-myosin interface as it is formed, we relied on current perspectives of the actin-myosin binding mechanism based on actin-myosin cryo-EM structures and myosin crystal structures [24,77]. Additionally, we examined the seven highest-resolution actomyosin cryo-EM structures containing a variety of actin and myosin isoforms to define what aspects of the binding mechanism are conserved or variable [24,74,80,83,84,85,88]. Previous analysis of functionally distinct myosin motors suggested that due to sequence heterogeneity, the actin-myosin interface is not conserved across myosin classes [114]. Our comparisons reveal that despite sequence differences, there are key regions of the actin surface that are used by all myosin isoforms. Instead of broadly different isoform-specific binding modes, the actin-myosin interface can be split into two interfaces. The first is a conserved interface that is seemingly common to all myosin and central to the actin-myosin binding mechanism, which is shown in cyan in Figure 1C. The second is a variable interface that establishes an isoform-specific binding mode that plays a role in distinguishing myosin function, as shown in yellow in Figure 1C. 

Because of their importance to actin binding, it is not surprising that the core interactions between actin and myosin are conserved. Phylogenetic studies report that current eukaryotic myosin motors are a result of gene duplications and subsequent functional fine-tuning [115,116]. As noted in ref [88], the conserved interface is retained despite the evolutionary distance between the isoforms compared here. This suggests that all myosin motors are constrained by the actin surfaces contacting the HLH motif and CM loop, but are free to adapt in an isoform-specific manner at the variable loops 2, 3, 4, and the activation loop. The constraint at the conserved interface is not strict, however. The cryo-EM structures reviewed here show that although the myosin sequence at the conserved interface may change modestly, the fixed actin residues continue to secure the motor using similar interactions. This arrangement between the unchanging actin surface and the adaptable myosin is expected to produce myosin motors that use the same core actin-binding regions but are “tuned” by structural changes at the variable interface or in the motor domain, modulating the kinetics of ATP hydrolysis and product release.

Although many of the effects of the variable interface are unknown, there are several examples of how the variable actin interface modulates myosin function. One example is the variable size of loop 2. As explained in the body of the text, myosin classes with very large loop 2 inserts, sometimes as large as 145 amino acids as in myosin IX, have an increased actin affinity throughout their ATPase cycle. It is through this affinity that these motors remain close to the actin surface regardless of nucleotide state, allowing them to become processive single-head motors. Another example of the variable interface impacting function is the interaction between loop 4, the actin surface, and tropomyosin. Some myosin motors, such as the unconventional myosin 1b, do not bind to actin filaments containing tropomyosin. This is most likely a reflection of the interaction with the variable loop 4. These examples show that the variable interface has the potential to affect actin-myosin characteristics. Much less is known about how the changes in loop 3, the activation loop, and at the tip of the CM loop affect motor function. More work needs to be completed to identify how each portion of the variable interface modulates actin-myosin activity.

While we have shown myosin sequence differences are important in isoform-specific function, actin isoform differences distant from the myosin binding site may also affect motor activity. For example, motility rates and ATPase activity for non-muscle myosin II isoforms, are α, β, or γ actin isoform dependent, even though the amino acid sequence at the actin-myosin interface is nearly identical [117]. Other studies have shown that the *Plasmodium falciparum* myosin, PfMyoA, has similar in vitro motility rates when paired with different actin isoforms, including PfActA1, skeletal actin, and smooth muscle actin [88]. The mechanisms underlying these actin isoform-specific differences/similarities are still unclear and warrant further structural characterization.

It is important to note that the comparisons of the actin surface bound to different myosin motors have been completed in only the rigor and strong-binding ADP states. Although the proposed binding mechanism describes the discrete binding steps of each myosin subdomain, experimental evidence of these intermediates is still missing. To get a more complete view of the rearrangements during actin-myosin binding, it will be necessary to visualize the actin-myosin complex trapped at each step of the ATPase cycle. For example, this could be accomplished by solving high-resolution actin-myosin structures with myosin trapped in intermediate conformations using nucleotide analogs in the place of ATP. Although these experiments would be difficult due to decreased actin-binding affinity, they would clarify how differences at the variable interface would affect the binding of actin-myosin intermediates and lead to functional differences. This information will be useful in predicting how motors function, designing myosin motors with specific properties and understanding how disease-causing mutations affect the actin-myosin interface and ATPase cycle.

## Data Availability

All data used in this review is publicly accessible. The structures analyzed in this study and their respective PDB IDs are provided in Supplementary Item Material Table S2.

## Supplementary Materials

The following are available online at https://www.mdpi.com/article/10.3390/biology10121221/s1, Supplementary Item Table S1: F-actin-binding Proteins Description and Supplementary Item Table S2: Summary of cryo-EM structures deposited in the PDB and Supplementary Item Table S3: Strength of the Actin-Myosin Interface Regions.
