# Supplementary material for: The Central Role of the F-Actin Surface in Myosin Force Generation
Source: Biology (Basel). 2021 Nov 23;10(12):1221. doi: 10.3390/biology10121221 (PMC8698748; doi:10.3390/biology10121221)
Supplement: Supplementary file 1 [file biology-10-01221-s001.zip › biology-1434526-supplementary.pdf]

Supplementary Information

The central role of the F-actin surface in myosin force generation

Matthew H. Doran and William Lehman

Supplementary Table S1: F-actin Binding Proteins Description

Protein Name	Function	Actin-Binding Domain	Actin Localization
Spectrin [1]	Cytoskeletal organization	CH domain	D-loop, SD1, SD2
Utrophin [2]	Anchors neuromuscular junction	CH domain	D-loop, SD1, SD2
Plastin [3]	F-actin bundling protein	CH domain	D-loop, SD1, SD2
LifeAct [4,5]	F-actin marker	-	D-loop
Cofilin [6]	F-actin severing	-	D-loop, SD1, SD2
MLCK [7]	Regulate smooth muscle contraction	-	SD1, SD4
Villin [8]	F-actin bundling protein	Headpiece Domain	-
Scruin [9]	F-actin cross-linker	-	SD1, SD2, SD3
Troponin [10]	Ca^{2+} dependent regulation of muscle contraction	-	-
Tropomyosin [11]	Ca^{2+} dependent regulation of muscle contraction	α/ β pseudorepeat	SD3, SD4
N-term Myosin Binding Protein C [12]	Modulates actin-myosin binding	-	SD1
Myosin [13]	Generating force on F-actin	-	D-loop, SD1, SD2, SD3
Calponin [14]	Modulates actin-myosin binding	CH domain	D-loop
Coronin [15]	Remodeling actin cytoskeleton	Beta-Propeller Domain	-
α-catenin [16]	Tethers cell-cell adhesion to cytoskeleton	-	D-loop, SD1, SD2, SD3
Metavinculin [16]	Anchoring actin to membrane	-	D-loop, SD1, SD2
Formin [17]	Remodeling of cytoskeleton	FH2	SD1, SD2, SD3, SD4
α-actinin [18]	Actin cross-linker in sarcomeres z band	CH domain	D-loop, SD1, SD2
Leiomodin [19]	F-actin cross-linker	WH2-domain	SD1, SD2
Tropomodulin [19]	Capping protein		SD1, SD2

Table S1: F-actin-binding Proteins. Summary of F-actin binding proteins. 20 F-actin binding proteins, their function, and localization on the actin filament have been tabulated to show their diversity of function. Notably, many F-actin binding proteins bind to the D-loop hydrophobic patch at the junction between two actin subunits.

Supplementary Table S2: Summary of cryo-EM structures deposited in the PDB.

PDB Code	EMDB Code	Resolution	Actin Species	Tropomyosin Species	Myosin Species	Isoform Name	Isoform Function	Bound State
6x5z* [20]	22067	4.2	Chicken ACTA1- P68139)	Human TPM1 - P09493	Bovine MYH7 - Q9BE39	β-cardiac muscle myosin II	Sarcomeric	Rigor
5jlh* [21]	8164	3.9	Human ACTG1 - P63261	Human TPM3 - P06753	Human MYH14 - Q7Z406)	Non-muscle myosin IIc (NM2c)	Cytoskeletal Motor	Rigor
7jh7* [22]	22335	3.8	Wild Boar ACTC1 - B6VNT8	Porcine TMP1 - P42639	Wild Boar MYH7 - P79293	β-cardiac muscle myosin II	Sarcomeric	Rigor
6c1d* [23]	7329	3.2	Rabbit ACTA1 - P68135	N/A	Rat Myo1b - Q05096	Unconventional myosin 1b (Myo1b)	Cytoskeletal Motor	ADP
6c1h* [23]	7331	3.9	Rabbit ACTA1 - P68135	N/A	Rat Myo1b - Q05096	Unconventional myosin 1b (Myo1b)	Cytoskeletal Motor	Rigor
6c1g* [23]	7330	3.8	Rabbit ACTA1 - P68135	N/A	Rat Myo1b - Q05096	Unconventional myosin 1b (Myo1b)	Cytoskeletal Motor	ADP
7aln* [24]	11818	3.8	*Plasmodium Falciparum* PfAct1 - Q8I4X0	N/A	*Plasmodium Falciparum* PfMyoA - Q8IDR3	PfMyoA	*Plasmodium* gliding motility	Rigor
6BNP* [25]	7116	4.6	Rabbit ACTA1 - P68135	N/A	Wild Boar MYO6 - Q29122	Unconventional myosin 6	(-) End Cytoskeletal Motor	Rigor
6BNQ* [25]	7117	5.5	Rabbit ACTA1 - P68135	N/A	Wild Boar MYO6 - Q29122	Unconventional myosin 6	(-) End Cytoskeletal Motor	ADP
5h53* [80]	6664	5.2	Rabbit ACTA1 - P68135	N/A	Rabbit MYH13 - Q9GJP9	Skeletal muscle myosin II	Sarcomeric	Rigor
6bih [26]	7100	6.0	Rabbit ACTA1 - P68135	N/A	Chicken MYH11 - P10587	Smooth muscle myosin II	Smooth Muscle Contraction	Rigor
4a7f [13]	1987	7.7	Rabbit ACTA1 - P68135	Rabbit TPM1 - P58772	Dictostelium MYOE - Q03479	Unconventional myosin IE	Cytoskeletal Motor	Rigor

Table S2: Summary of cryo-EM structures deposited in the PDB. All currently published actin-myosin cryo-EM structures are tabulated in order to compare relevant information. The resolution is reported as listed in the RCSB Protein Data Bank (PDB)/ EM Data Bank (EMDB) pages. Species Uniprot sequences were taken from the PDB. The seven isoforms, which report resolution high enough to define the protein backbone (<5.2Å) compared **Figures 4-7** are listed with a star next to their PDB code. The citation associated with the PDB entries are also listed in the same column. Since 6X5Z and 7JH7 both contain β-cardiac muscle myosin II isoforms that are nearly identical at the actin-myosin interface, only one is used for comparisons.

Supplementary Table S3: Strength of the Actin-Myosin Interface Regions

Myosin Isoform	Cardiomyopathy Loop	HLH Motif	Loop 3	Loop 4
**β-cardiac muscle myosin II**	Buried Surface: 353.3 $Å^2$<br>ΔG: -5.0 kcal/mole	Buried Surface: 549.9 $Å^2$<br>ΔG: -9.8 kcal/mole	No interaction	Buried Surface: 137.4 $Å^2$<br>ΔG: -0.2 kcal/mole
**Non-muscle myosin IIc (NM2c)**	Buried Surface: 405.2 $Å^2$<br>ΔG: -4.1 kcal/mole	Buried Surface: 639.2 $Å^2$<br>ΔG: -6.6 kcal/mole	Buried Surface: 163.1 $Å^2$<br>ΔG: -0.8 kcal/mole	Buried Surface: 103.3 $Å^2$<br>ΔG: 0.4 kcal/mole
**Plasmodium Falciparum PfMyoA**	Buried Surface: 339.1 $Å^2$<br>ΔG: -4.4 kcal/mole	Buried Surface: 605.3 $Å^2$<br>ΔG: -7.9 kcal/mole	Buried Surface: 242.2 $Å^2$<br>ΔG: -1.8 kcal/mole	Buried Surface: 182.3 $Å^2$<br>ΔG: -2.0 kcal/mole
**Unconventional myosin 6**	Buried Surface: 334.7 $Å^2$<br>ΔG: -5.0 kcal/mole	Buried Surface: 399.6 $Å^2$<br>ΔG: -4.5 kcal/mole	Buried Surface: 318.2 $Å^2$<br>ΔG: 2.3 kcal/mole	Buried Surface: 208.0 $Å^2$<br>ΔG: 0.5 kcal/mole
**Unconventional myosin 1b (Myo1b)**	Buried Surface: 425.5 $Å^2$<br>ΔG: -3.6 kcal/mole	Buried Surface: 484.5 $Å^2$<br>ΔG: -4.8 kcal/mole	Buried Surface: 331.9 $Å^2$<br>ΔG: -1.0 kcal/mole	Buried Surface: 167.5 $Å^2$<br>ΔG: -1.0 kcal/mole
**Skeletal muscle myosin II**	Buried Surface: 239.1 $Å^2$<br>ΔG: -4.1 kcal/mole	Buried Surface: 420.0 $Å^2$<br>ΔG: -6.5 kcal/mole	Buried Surface: 68.1 $Å^2$<br>ΔG: 0.5 kcal/mole	Buried Surface: 38.9 $Å^2$<br>ΔG: 0.6 kcal/mole

Table S3: Tabulating the strength of each region involved in actin-myosin binding. The buried surface area as well as the free energy of dissociation (ΔG) for each actin-myosin interface in the six isoforms compared in this review. These values were calculated in the PDB web application, PISA. Each actin-myosin region was extracted from their original pdb files and run through the PISA web app. The tabulated values reflect a trend where the HLH motif is the largest and strongest interface for each isoform (except unconventional myosin 6, which has an unusually large CM loop), followed by the CM loop. The variable loops, Loop 3 and Loop 4 make a comparatively smaller contribution to binding and each isoform has vastly different values for these regions. The activation loop was not shown due to its very small interface with the actin filament. Loop 2 was not included because it does not appear in the β-cardiac muscle myosin II, Non-muscle myosin IIc (NM2c), and Skeletal muscle myosin II cryo-EM densities.

Supplementary Citations:

1. Djinovic-Carugo, K.; Gautel, M.; Ylänne, J.; Young, P. The Spectrin Repeat: A Structural Platform for Cytoskeletal Protein Assemblies. *FEBS Lett* **2002**, *513*, 119–123, doi:10.1016/s0014-5793(01)03304-x.
2. Lin, A.Y.; Prochniewicz, E.; James, Z.M.; Svensson, B.; Thomas, D.D. Large-Scale Opening of Utrophin's Tandem Calponin Homology (CH) Domains upon Actin Binding by an Induced-Fit Mechanism. *Proc Natl Acad Sci U S A* **2011**, *108*, 12729–12733, doi:10.1073/pnas.1106453108.
3. Morley, S.C. The Actin-Bundling Protein L-Plastin: A Critical Regulator of Immune Cell Function. *Int J Cell Biol* **2012**, *2012*, 935173, doi:10.1155/2012/935173.
4. Belyy, A.; Merino, F.; Sitsel, O.; Raunser, S. Structure of the Lifeact–F-Actin Complex. *PLOS Biology* **2020**, *18*, e3000925, doi:10.1371/journal.pbio.3000925.
5. Kumari, A.; Kesarwani, S.; Javoor, M.G.; Vinothkumar, K.R.; Sirajuddin, M. Structural Insights into Actin Filament Recognition by Commonly Used Cellular Actin Markers. *EMBO J* **2020**, *39*, e104006, doi:10.15252/embj.2019104006.
6. Tanaka, K.; Takeda, S.; Mitsuoka, K.; Oda, T.; Kimura-Sakiyama, C.; Maéda, Y.; Narita, A. Structural Basis for Cofilin Binding and Actin Filament Disassembly. *Nat Commun* **2018**, *9*, 1860, doi:10.1038/s41467-018-04290-w.
7. Hatch, V.; Zhi, G.; Smith, L.; Stull, J.T.; Craig, R.; Lehman, W. Myosin Light Chain Kinase Binding to a Unique Site on F-Actin Revealed by Three-Dimensional Image Reconstruction. *J Cell Biol* **2001**, *154*, 611–618, doi:10.1083/jcb.200105079.
8. Meng, J.; Vardar, D.; Wang, Y.; Guo, H.-C.; Head, J.F.; McKnight, C.J. High-Resolution Crystal Structures of Villin Headpiece and Mutants with Reduced F-Actin Binding Activity. *Biochemistry* **2005**, *44*, 11963–11973, doi:10.1021/bi050850x.
9. Owen, C.; DeRosier, D. A 13-A Map of the Actin-Scruin Filament from the Limulus Acrosomal Process. *J Cell Biol* **1993**, *123*, 337–344, doi:10.1083/jcb.123.2.337.
10. Yamada, Y.; Namba, K.; Fujii, T. Cardiac Muscle Thin Filament Structures Reveal Calcium Regulatory Mechanism. *Nat Commun* **2020**, *11*, 153, doi:10.1038/s41467-019-14008-1.
11. Lehman, W.; Li, X.; Kiani, F.A.; Moore, J.R.; Campbell, S.G.; Fischer, S.; Rynkiewicz, M.J. Precise Binding of Tropomyosin on Actin Involves Sequence-Dependent Variance in Coiled-Coil Twisting. *Biophysical Journal* **2018**, *115*, 1082–1092, doi:10.1016/j.bpj.2018.08.017.
12. Risi, C.; Belknap, B.; Forgacs-Lonart, E.; Harris, S.P.; Schröder, G.F.; White, H.D.; Galkin, V.E. N-Terminal Domains of Cardiac Myosin Binding Protein C Cooperatively Activate the Thin Filament. *Structure* **2018**, *26*, 1604-1611.e4, doi:10.1016/j.str.2018.08.007.
13. Behrmann, E.; Müller, M.; Penczek, P.A.; Mannherz, H.G.; Manstein, D.J.; Raunser, S. Structure of the Rigor Actin-Tropomyosin-Myosin Complex. *Cell* **2012**, *150*, 327–338, doi:10.1016/j.cell.2012.05.037.
14. Hartwig, J.H. Actin-Binding Proteins 1: Spectrin Superfamily. *Protein Profile* **1994**, *1*, 706–778.
15. de Hostos, E.L.; Bradtke, B.; Lottspeich, F.; Guggenheim, R.; Gerisch, G. Coronin, an Actin Binding Protein of Dictyostelium Discoideum Localized to Cell Surface Projections, Has Sequence Similarities to G Protein Beta Subunits. *EMBO J* **1991**, *10*, 4097–4104.
16. Mei, L.; Espinosa de los Reyes, S.; Reynolds, M.J.; Leicher, R.; Liu, S.; Alushin, G.M. Molecular Mechanism for Direct Actin Force-Sensing by α-Catenin. *eLife* **2020**, *9*, e62514, doi:10.7554/eLife.62514.
17. Courtemanche, N. Mechanisms of Formin-Mediated Actin Assembly and Dynamics. *Biophys Rev* **2018**, *10*, 1553–1569, doi:10.1007/s12551-018-0468-6.

18. Galkin, V.E.; Orlova, A.; Salmazo, A.; Djinovic-Carugo, K.; Egelman, E.H. Opening of Tandem Calponin Homology Domains Regulates Their Affinity for F-Actin. *Nat Struct Mol Biol* **2010**, *17*, 614–616, doi:10.1038/nsmb.1789.
19. Boczkowska, M.; Rebowski, G.; Kremneva, E.; Lappalainen, P.; Dominguez, R. How Leiomodin and Tropomodulin Use a Common Fold for Different Actin Assembly Functions. *Nat Commun* **2015**, *6*, 8314, doi:10.1038/ncomms9314.
20. Doran, M.H.; Pavadai, E.; Rynkiewicz, M.J.; Walklate, J.; Bullitt, E.; Moore, J.R.; Regnier, M.; Geeves, M.A.; Lehman, W. Cryo-EM and Molecular Docking Shows Myosin Loop 4 Contacts Actin and Tropomyosin on Thin Filaments. *Biophysical Journal* **2020**, doi:10.1016/j.bpj.2020.07.006.
21. von der Ecken, J.; Müller, M.; Lehman, W.; Manstein, D.J.; Penczek, P.A.; Raunser, S. Structure of the F–Actin–Tropomyosin Complex. *Nature* **2015**, *519*, 114–117, doi:10.1038/nature14033.
22. Risi, C.; Schäfer, L.U.; Belknap, B.; Pepper, I.; White, H.D.; Schröder, G.F.; Galkin, V.E. High-Resolution Cryo-EM Structure of the Cardiac Actomyosin Complex. *Structure* **2021**, *29*, 50-60.e4, doi:10.1016/j.str.2020.09.013.
23. Mentes, A.; Huehn, A.; Liu, X.; Zwolak, A.; Dominguez, R.; Shuman, H.; Ostap, E.M.; Sindelar, C.V. High-Resolution Cryo-EM Structures of Actin-Bound Myosin States Reveal the Mechanism of Myosin Force Sensing. *Proc Natl Acad Sci U S A* **2018**, *115*, 1292–1297.
24. Robert-Paganin, J.; Xu, X.-P.; Swift, M.F.; Auguin, D.; Robblee, J.P.; Lu, H.; Fagnant, P.M.; Krementsova, E.B.; Trybus, K.M.; Houdusse, A.; et al. The Actomyosin Interface Contains an Evolutionary Conserved Core and an Ancillary Interface Involved in Specificity. *Nat Commun* **2021**, *12*, 1892, doi:10.1038/s41467-021-22093-4.
25. Gurel, P.S.; Kim, L.Y.; Ruijgrok, P.V.; Omabegho, T.; Bryant, Z.; Alushin, G.M. Cryo-EM Structures Reveal Specialization at the Myosin VI-Actin Interface and a Mechanism of Force Sensitivity. *eLife* **2017**, *6*, e31125, doi:10.7554/eLife.31125.
26. Banerjee, C.; Hu, Z.; Huang, Z.; Warrington, J.A.; Taylor, D.W.; Trybus, K.M.; Lowey, S.; Taylor, K.A. The Structure of the Actin-Smooth Muscle Myosin Motor Domain Complex in the Rigor State. *J Struct Biol* **2017**, *200*, 325–333, doi:10.1016/j.jsb.2017.10.003.
